# Molecular evidence of parallel evolution in a cyanophage

**DOI:** 10.1371/journal.pone.0281537

**Published:** 2023-02-09

**Authors:** Jesslyn Tjendra, Julia E. Storesund, Håkon Dahle, Ruth-Anne Sandaa, Selina Våge

**Affiliations:** 1 Department of Biological Sciences, University of Bergen, Bergen, Norway; 2 Institute of Marine Research, Bergen, Norway; University of Bath, UNITED KINGDOM

## Abstract

Antagonistic interactions between bacteriophage (phage) and its bacterial host drives the continual selection for resistance and counter-defence. To date, much remains unknown about the genomic evolution that occurs as part of the underlying mechanisms. Such is the case for the marine cyanobacteria *Synechococcus* and viruses (cyanophages) that infect them. Here, we monitored host and phage abundances, alongside genomic changes to the phage populations, in a 500-day (~55 bacterial generations) infection experiment between *Synechococcus* sp. WH7803 and the T4-type cyanophage S-PM2d, run parallel in three replicate chemostats (plus one control chemostat). Flow cytometric count of total abundances revealed relatively similar host-phage population dynamics across the chemostats, starting with a cycle of host population collapse and recovery that led to phases of host-phage coexistence. Whole-genome analysis of the S-PM2d populations detected an assemblage of strongly selected and repeatable genomic changes, and therefore parallel evolution in the phage populations, early in the experiment (sampled on day 39). These consisted mostly of non-synonymous single-nucleotide-polymorphisms and a few instances of indel, altogether affecting 18 open-reading-frames, the majority of which were predicted to encode virion structures including those involved in phage adsorption onto host (i.e., baseplate wedge, short tail fibre, adhesin component). Mutations that emerged later (sampled on day 500), on the other hand, were found at a larger range of frequencies, with many lacking repeatability across the chemostats. This is indicative of some degree of between-population divergence in the phage evolutionary trajectory over time. A few of the early and late mutations were detected within putative auxiliary metabolic genes, but these generally occurred in only one or two of the chemostats. Less repeatable mutations may have higher fitness costs, thus drawing our attention onto the role of trade-offs in modulating the trajectory of a host-phage coevolution.

## Introduction

Infection pressure from bacteriophage (phage) selects for resistant bacterial host, which in turn exerts selection for phage that overcomes host defence, and so on. Through this reciprocal and continual selection for resistance and counter-defence, the host and phage interact and coevolve antagonistically [1,2]. This ‘arms race’ dynamics was first described in the Red Queen hypothesis [3] and has been proposed and demonstrated to be a mechanism driving genotypic and phenotypic diversity [1,4–6].

Microbial populations are ideal model systems for (co)evolution studies [1]. In a landmark study using *Escherichia coli* and the T-series phages, Lenski & Levin [7] formally presented mutational events as both a driver and constraint acting on the host-phage coevolution. Long-term antagonistic coevolution was first observed in ~400 (bacterial) generations of co-cultivation and persistent cycles of resistance and counter-defence between *Pseudomonas fluorescens* SBW25 and the phage SBW25ɸ2 [1]. The study provided evidence for directional selection in the coevolution towards wider resistance and infectivity ranges, as well as substantial divergence amongst replicate populations. The potential for extensive antagonistic coevolution in a marine host-phage system was first demonstrated between the cyanobacterium *Synechococcus* sp. WH7803 and the lytic cyanophage RIM8 [8]. Similarly, the emergence of multiple, distinct phenotypes was observed both in the host and the phage, attributable to their antagonistic interactions. The study also identified some of the genetic constituents linked to the observed phenotypes, including a putative gene in RIM8 of unknown function, non-conserved even amongst the cyanophages, yet particularly subjected to strong selection. This finding, together with the absence of detected mutation within homologs to known viral tail fiber genes, contributed to their conclusion that the genetic mechanisms driving *Synechococcus*-RIM8 coevolution may be distinct from those previously characterized for *E*. *coli* and *P*. *fluorescens* [5,6].

The molecular mechanisms behind a host-phage coevolution, and the interplay between deterministic and stochastic processes in this context, have remained enigmatic. At the same time, continual advances in modern sequencing technologies have facilitated major progresses in this field, allowing the reconstruction of evolutionary events at an unprecedented level of detail. Extending these lines of research, we sought a population genomics approach to characterize the underlying genotype and repeatability of the cyanophage S-PM2d evolution as it infected and coexisted with its host *Synechococcus* sp. WH7803. *Synechococcus* species are virtually ubiquitous in the marine environment. They serve ecologically important functions as primary producers, especially in the oligotrophic regions of the oceans [9,10]. As part of the marine microbial loop, infection and lysis of cyanobacteria by viruses (cyanophages) is also integral to marine ecosystem functioning by shunting photosynthetically fixed carbon back to the pool of dissolved organic matter [11,12]. The lytic cyanophage S-PM2d is a myovirus carrying double-stranded DNA of size 186,736 bp [13,14]. S-PM2d genome carries 211 open reading frames (ORFs), amongst which some are homologs to cyanobacterial genes (incl. auxiliary metabolic genes). These include the putative genes *psbA*, *psbD* and *hli*, encoding key components of the photosynthetic reaction centre PSII, the D1 and D2 proteins, and a high-light inducible protein (respectively). It has been proposed that their presence may serve to ensure continuation of photosynthesis in infected host cells and thus energy provision for phage replication [15].

In our present study, we monitored the host-phage population abundances through 500 days (~55 bacterial generations) of co-cultivation run parallel in three replicate chemostats (plus one control chemostat with only *Synechococcus*). Phage mutations that emerged early on (sampled on day 39) and later (sampled on day 500) in the experiment were identified. In contrast to the individual-level analysis of select genes (as well as whole-genomes) in the previous study on *Synechococcus*-RIM8 [8], we inspected whole-genome sequences from the phage populations to capture and assemble a broad overview of the genomic changes. Comparisons were also made across the three phage populations (replicate chemostats) to address the question of the extent to which the phage genomic evolution was repeatable. This gave us insights into the predictability of diversity changes at temporal scale, as well as the potential role of fitness costs of mutations (i.e., trade-offs) in the evolutionary trajectory.

## Materials and methods

### Host and phage strains

Axenic cultures of *Synechococcus* sp. WH7803 and phage S-PM2d (*Myoviridae*) were kindly provided by Dr. Martha Clokie (University of Leicester, UK). *Synechococcus* strain was maintained at 23°C under continuous light (20–23 μE m^-2^ s^-1^) on artificial seawater medium (ASW) containing per L: 25 g NaCl; 2 g MgCl_2_ x 6H_2_O; 0.5 g KCl; 0.75 g NaNO_3_; 0.002 g K_2_HPO_4_ x 3H_2_O; 3.5 g MgSO_4_ x 7H_2_O; 0.5 g CaCl_2_ x 2H_2_O; 1.1 g Trizma buffer (Sigma), and 1 mL trace metal solution containing per L: 2.86 g 1H_3_BO_3_; 1.81 g MnCl_2_ x 4H_2_O; 0.222 g ZnSO_4_x 7H_2_O; 0.39 g Na2MoO_4_ x 2H_2_O; 0.008 g CuSO_4_ x 5H_2_O; 0.0049 g Co(NO_3_)_2_ x 6H_2_O; 3.0 g FeCl_3_ x 6H_2_O and 0.5 g EDTA, as described in [16,17] but with reduced phosphate concentration.

Clonal strain of the phage S-PM2d was cultured by picking a single plaque from a plaque assay and then re-suspending it in a liquid culture of *Synechococcus* sp. WH7803. The culture was spun down after host-lysis at 6000 rpm for 20 min to remove cell debris. The supernatant containing the phage particles (lysate) was then filtered (0.22 μm) to remove any remaining cells and stored at 4°C until addition into the chemostats.

### Chemostat set-up and sampling

Non-clonal cultures of *Synechococcus* sp. WH7803 were established in four 500 mL glass round bottom flasks receiving a continuous in-flow of ASW with phosphate concentration of 10 μM. Media was supplied from the same reservoir to all chemostats. Generation time was optimized by adjusting the in-flow of fresh media to 37 mL day^-1^, giving generation times of 8.5–9.5 days. This was done to make the cells phosphate-limited, yet still capable of maintaining a cell concentration of approximately 3–6 x 10^7^ cells mL^-1^. The cultures were allowed to stabilize to dilution rates and nutrient availability in the chemostats, thus reaching and staying at steady-state, for approx. four weeks prior to addition of phage. A clonal strain of phage S-PM2d was added to three of the four chemostats in a multiplicity of infection (MOI) of ~0.01. All four chemostats were run in parallel, maintained at 23°C and under continuous light (20–23 μE m^-2^ s^-1^) for 500 days (~55 generations) following phage addition.

Chemostat cultures were sampled periodically throughout the experiment for flow cytometric monitoring of *Synechococcus* and S-PM2d abundances. Samples for flow cytometric counts were fixed with 0.5% v/v glutaraldehyde, incubated at 4°C for 30 min, flash-frozen in liquid nitrogen, and stored at -80°C until further processing. On days 39 (‘early’) and 500 (‘late’) following phage addition (day 0), samples of the chemostat cultures were collected for phage DNA extraction and sequencing.

### Flow cytometry (FCM)

A FACSCalibur^™^ flow cytometer (Becton Dickinson) was used to enumerate *Synechococcus* and S-PM2d abundances in the chemostats, based on protocols described in [18–20]. Samples were diluted 10- to 10^4^-fold in TE buffer (10:1, pH 8), stained with SYBR Green I, incubated in the dark–first at room temperature and then at 80°C for 10 min each–and then run in the flow cytometer for 1 min at flow rates of ca. 30–35 μL min^-1^ and counting events of ca. 100–1000 s^-1^. *Synechococcus* and S-PM2d were distinguished from one another based on their side scatter versus green fluorescence signal.

### Phage DNA extraction and sequencing

50 mL of the chemostat culture was centrifuged at 6000 rpm for 20 min to discard *Synechococcus* cells. The supernatant was filtered (0.22 μm) to remove remaining cells and then ultracentrifuged (25000 rpm in a Beckman Optima L-90K Ultracentrifuge) to pellet the phage particles. The phage pellet was re-suspended in SM buffer and stored at -80°C until DNA extraction.

Phage particles were lysed using the proteinase K/sodium dodecyl sulphate (SDS) method described in [21]. DNA purification was performed using the Zymo Research Genomic DNA Clean & Concentrator-10 kit. Illumina library preparation and sequencing was carried out at the Norwegian Sequencing Centre (https://www.sequencing.uio.no), following the TruSeq^™^ protocol (Illumina, Inc., San Diego, USA) and MiSeq 300 bp paired-end sequencing (Illumina, Inc., San Diego, USA).

### Genomic analysis

Cutadapt v.1.15 [22] was used to trim off Illumina TruSeq adapters (R1: AGATCGGAAGAGCACACGTCTGAACTCCAGTCA; R2: AGATCGGAAGAGCGTCGTGTAGGGAAAGAGTGT) from all raw sequence reads, and then to remove reads that were shorter than 20 bp. Quality assessment of the sequence reads using FastQC v.0.11.9 revealed that forward (R1) reads generally had higher Phred scores than reverse (R2) reads for all samples. Only the forward (R1) reads were therefore used for all alignments in this analysis. Using Bowtie 2 v.2.3.5 [23], reads from the original (clonal) phage strain were mapped to the EMBL-deposited S-PM2d genome (Acc. No. LN828717.1) as reference sequence. A total of 1,234,064 forward (R1) reads aligned exactly once to yield a mean (sequencing) depth of 1654 ±529. Samtools v.1.10 [24] was used for downstream processing (sorting and indexing) of the SAM alignment file. A consensus sequence was drawn from the alignment using Bcftools v.1.10.2 [25]. This was done by calling variants on the alignment, with the mpileup option ‘-d’ set to 0 to remove the depth limit and the call options ‘-vmO z’ on. Indels in the compressed VCF (variant call format) file were normalized, and adjacent indels within 5 bp were filtered away. The compressed VCF file was then quality-filtered and indexed, before the VCF variants were applied to the reference S-PM2d genome to generate a consensus sequence.

The consensus sequence served as reference sequence for Bowtie 2 alignment of reads from day-39 and -500 samples. Bcftools was again used to call variants in these alignments from that of the original phage strain. The resulting list of variants were manually checked through alignment visualisation on the Integrative Genomics Viewer (IGV) [26], and individually assessed as potential mutations. Only those found at frequencies of ≥ 20% were selected for further analysis to avoid including sequencing error as mutation. Variants found above the frequency threshold but at low sequencing depth were also excluded.

## Results

### Early in the *Synechococcus*–S-PM2d coexistence

**Fig 1:**
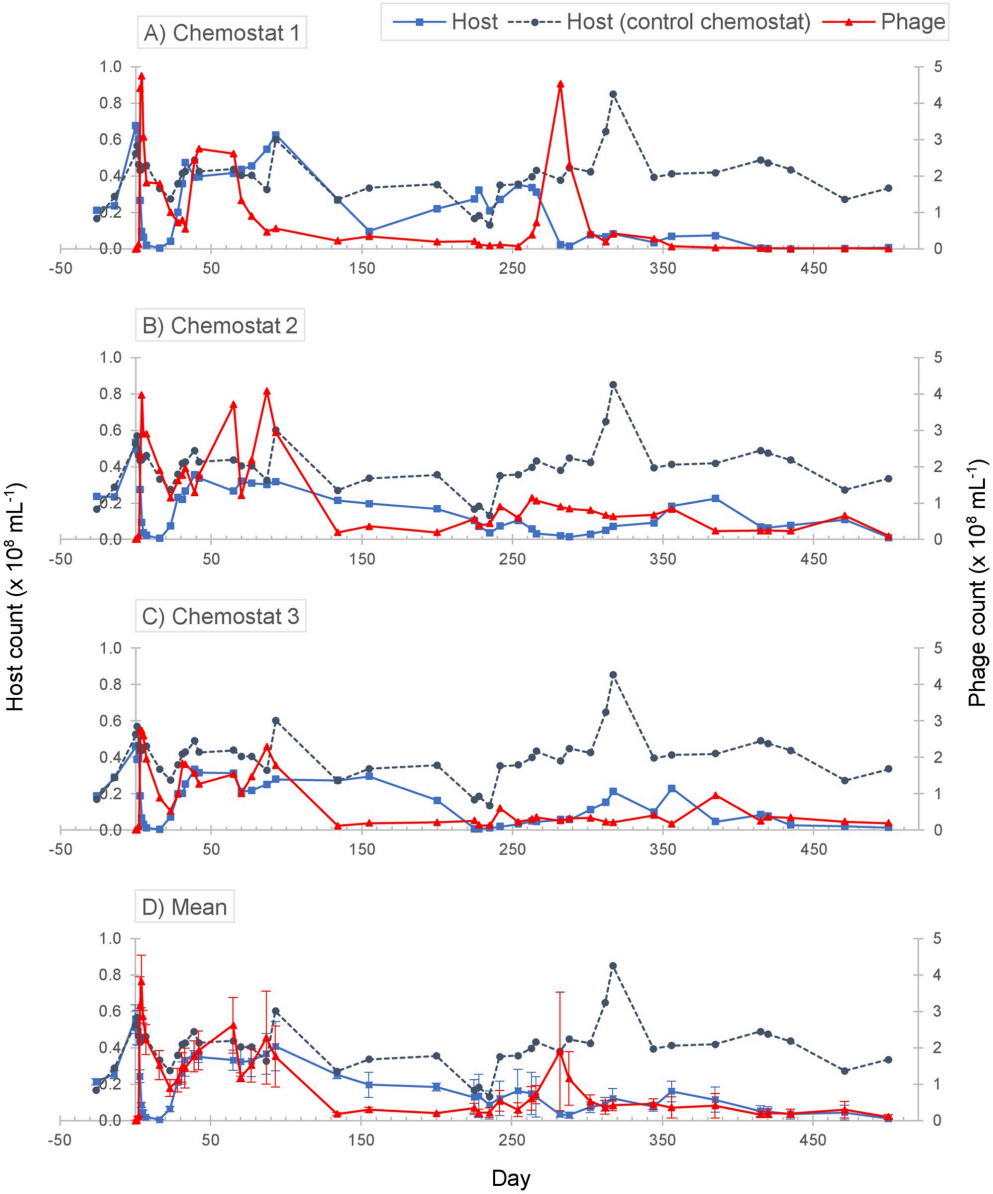
Host *Synechococcus* sp. WH7803 and cyanophage S-PM2d population dynamics. Abundances, specifically the flow cytometric (FCM) counts in 10^8^ per mL, of the host (◼) and phage (▲) over the course of a 500-day (~55 bacterial generations) infection experiment, run parallel in three replicate chemostats 1–3 (panels A-C). Mean abundances across the three replicate chemostats (n = 3; error bar = SEM) are plotted in panel D. For reference, *Synechococcus* abundance in the control (phage-free) chemostat (⚫) is plotted in all four panels. Under steady-state throughout the experiment, the abundance of *Synechococcus* in the control chemostat averaged at 4.01 ±1.28 x 10^7^ mL^-1^. Phage was added into the chemostat cultures on day 0, and the phage populations were sampled for genomic analysis on days 39 (‘early’) and 500 (‘late’) of the experiment.

Considering a steady-state average throughout the experiment, the abundance of *Synechococcus* in the control chemostat amounted to 4.01 ±1.28 x 10^7^ mL^-1^ (n = 44). The host-phage population dynamics across the three replicate chemostats were comparable over the course of the first 39 days (~4 bacterial generations) following phage addition (Fig 1). S-PM2d immediately triggered a collapse in *Synechococcus* population down to ca. 1% of the initial and control population abundances, averaging at 4.83 ±1.31 x 10^5^ mL^-1^ (n = 3), within 16 days (~2 generations) of phage infection. Thereafter, all three host populations recovered and by day 31 (~3 generations), their abundances did not appear to differ from that of the control population. The relatively quick collapse and recovery of *Synechococcus* indicates that the population was initially dominated by an ecotype susceptible to infection by S-PM2d, but cells that were intrinsically phage-resistant were likely already present. As the susceptible cells lysed, the resistant ones could then use the available resources to grow and repopulate the culture. During this period, S-PM2d in the three chemostats proliferated by four orders of magnitude and then fluctuated around the densities of 10^8^ mL^-1^. The coupling of these observations on the host and phage populations indicates that by day 39, the host-phage system in all three replicate chemostats had entered a phase of stable coexistence whereby phage production appeared to continue despite dominance of resistant host.

**Fig 2:**
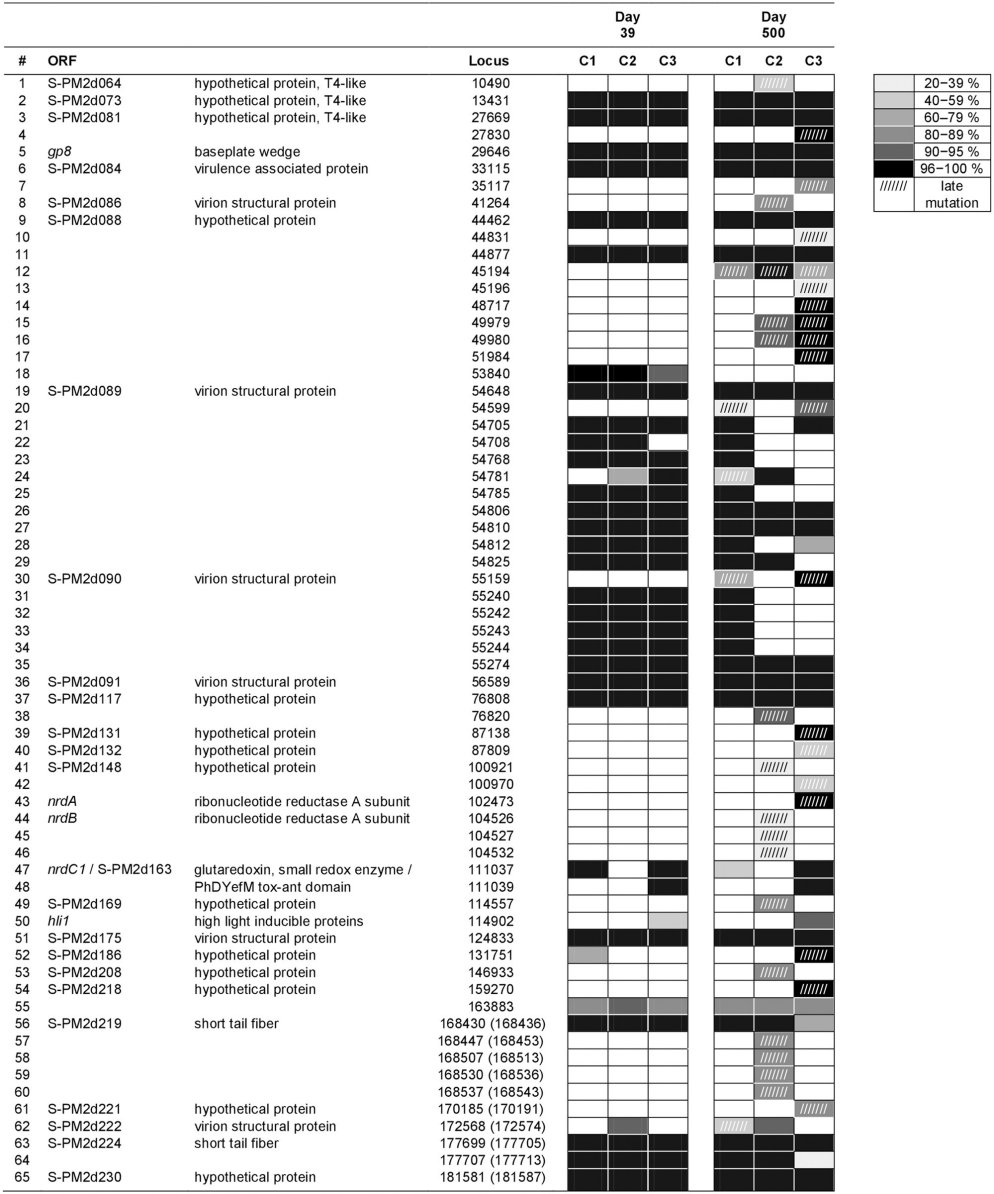
Overview of the genomic changes to the cyanophage S-PM2d populations. The loci and frequencies of mutations detected in the cyanophage S-PM2d populations across three replicate chemostats (C 1–3), 39 and 500 days after infecting the host *Synechococcus* sp. WH7803. Aside from #2 and #18, all the mutations are non-synonymous. The intensity of the shaded cell indicates the frequency (i.e. % of the corresponding sequencing depth) at which the mutation was found. Unshaded cell = absence of mutation; cell with slashes = late mutation detected only on day 500; ORF = open reading frame. In general, the early mutations (detected on day 39) are present in all three cyanophage populations and at very high frequencies, whereas the late mutations (detected on day 500) are less repeatable across the populations and are found at a larger range of frequencies.

**Fig 3:**
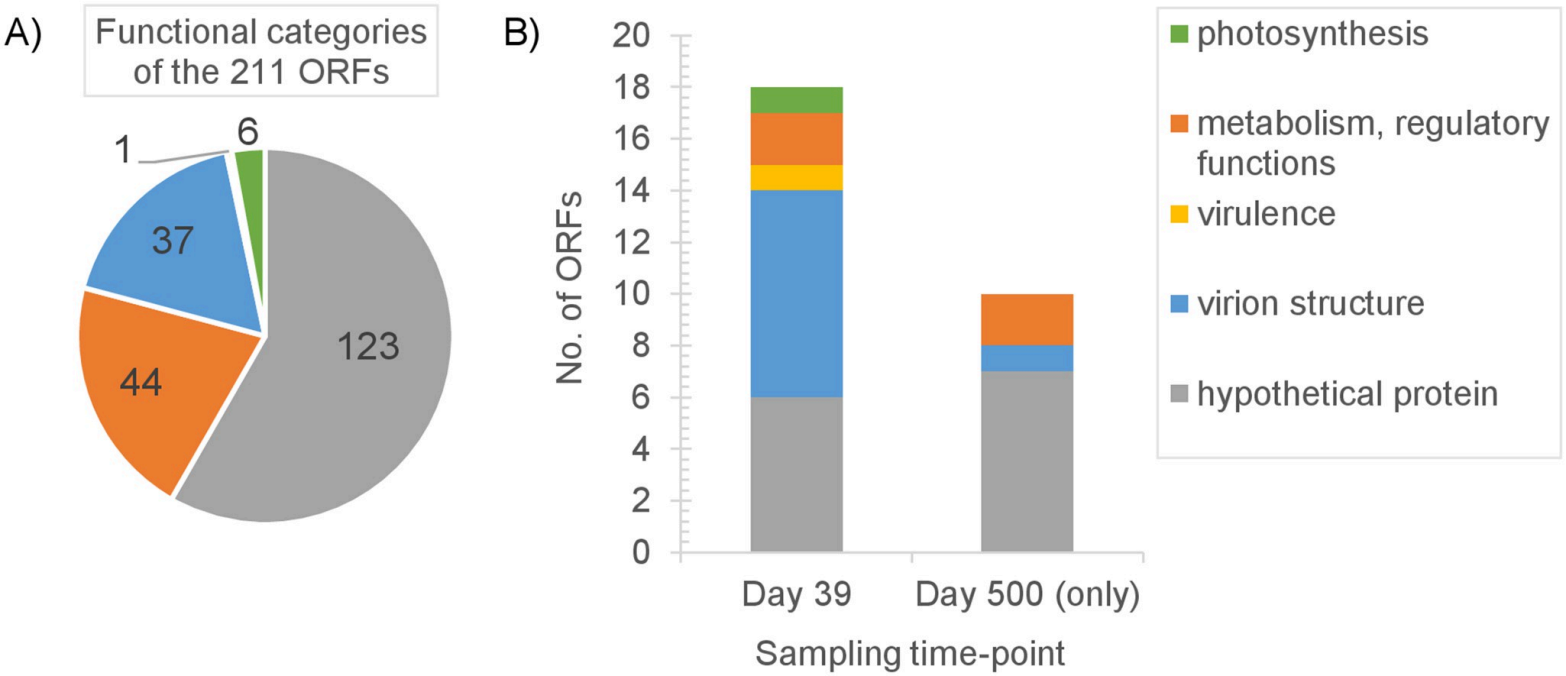
Cyanophage S-PM2d open reading frames (ORFs). The numbers and functional associations of S-PM2d ORFs A) in total; B) carrying non-synonymous mutations found on days 39 and 500 following infection of the host *Synechococcus* sp. WH7803.

A total of 33 non-synonymous mutations were identified from whole-genome sequencing and analysis of the three S-PM2d populations sampled on day 39. Most of these mutations were found at the frequency of 100% (i.e., across 100% of the bases or reads mapped to the particular locus in the alignment) and, also, across all three replicates (Fig 2). This suggests strong selective advantages to carrying these phage mutations. Two additional synonymous mutations were detected (mutations #2 and #18 in Fig 2; refer to S1-2 Table in S1 Appendix for details). Apart from three instances of indel (mutations #50, #55, #62), the non-synonymous mutations were single nucleotide polymorphisms (SNPs), altogether affecting 18 (out of a total of 211; Fig 3A) open reading frames (ORFs). The majority (44%) of these affected ORFs encodes structural proteins (Fig 3B), including the baseplate wedge (*gp8*) and short tail fiber (S-PM2d219, S-PM2d224) of the virion (mutations #5, #56, #63). One notable example is the SNP within the ORF S-PM2d175 (mutation #51). S-PM2d175 is part of a contiguous block of genes that potentially encode components of adhesin, which are phage proteins directly involved in recognition and adhesion to the host [13]. A nucleotide substitution from cytosine to adenine (C→A) at locus 124,833 conferred an amino acid change from aspartic acid to glutamic acid (Asp→Glu). In the original phage strain, cytosine was found at the frequency of 62% of the 1650 bases or reads aligned to that locus. By day 39 into the host-phage coexistence, 100% of the bases found at that locus in the alignment was adenine for all three replicates, at sequencing depths of 3197, 2733, and 2693 for chemostats 1, 2, and 3 respectively.

**Fig 4:**
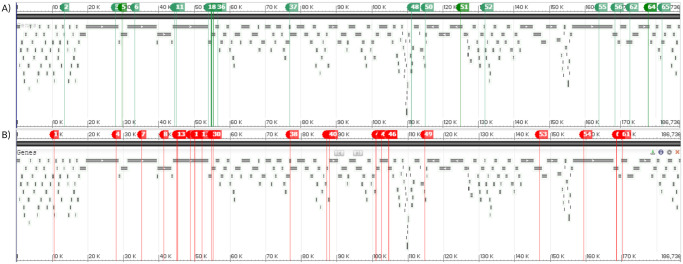
Locations of the mutations within the cyanophage S-PM2d genome organization. A) 33 non-synonymous and 2 synonymous early mutations (green markers) detected in the cyanophage populations on day-39 of infection experiment. B) 30 non-synonymous late mutations (red markers) detected on day-500. Numbers on markers correspond to the numbering of mutations in Fig 2. Image generated using the NCBI Sequence Viewer version 3.46 [27].

A considerable fraction (33%) of the affected ORFs are hypothetical proteins with homology to other bacterial and phage proteins but with unknown functions (Fig 3B). S-PM2d081 (mutation #3 in Fig 2 and S1-2 Table in S1 Appendix) and S-PM2d088 (mutations #9 and #11), in particular, are clustered in proximity to one another and are exceptionally large (3048 and 3338 amino acids, respectively (Fig 4A). So was S-PM2d218 (mutation #54), with its protein sequence of 3779 amino acids (aa). Other ORFs encode proteins associated with virulence (S-PM2d084; mutation #6), host metabolic (*nrdC1*, S-PM2d163; mutations #47 and #48) and photosynthetic activities (*hli1*; mutation #50). Two mutations occurred within a shared region between two overlapping metabolic genes. These were the glutaredoxin gene *nrdC1* and S-PM2d163 encoding a PhDYefM tox-ant domain. These mutations, however, were not found across all three replicates. Only the phage population in chemostats 1 and 3 carried one or both of them (Fig 2). So was the case for the mutation in the gene *hli1*, encoding a high-light inducible protein. It was a single nucleotide insertion that would have caused a shift in the downstream reading frame, and which was found to occur in chemostat 3 only. Furthermore, the mutation was found in only 41% of the 2646 reads aligned to that locus. Given that the frame shift would cause potentially extensive alterations to the polypeptide and functionality, it is plausible that this mutation would exert a heavier fitness cost on the phage.

A further look into the sequence scaffold for the original phage strain revealed that the frequencies of the initial nucleotides for seven out of the 33 non-synonymous mutations were notably lower than 100%, with the mutations having had emerged already at this point. Consequently, this has the implication that some of the early mutations were, in principle, alternative alleles rather than newly acquired mutations. These mutant alleles were present at low frequencies, although a few of them reached up to 49% (see S1-2 and S1-3 Tables in S1 Appendix for details). For instance, using the previous example of the gene encoding the adhesin component (S-PM2d175; mutation #51; Fig 2 and S1-2 Table in S1 Appendix), the original phage strain carried two alternative alleles at the particular locus, with the nucleotide cytosine being the dominant allele at 62% (of sequencing depth 1650) followed by adenine at 38%. 39 days post-infection, the frequency of the allele adenine independently shifted to 100% for all three phage populations. This shift, nonetheless, signifies strong positive selection of the mutant allele over the original one. The occurrence of these alternative alleles in the clonal phage population informed us of the acquisition of mutations and variability even during the process of phage lysate preparation itself, despite starting out from a single plaque and efforts to keep the lysate as isogenic as possible prior to infection. This was perhaps inevitable in practice, given the multitude of infection cycles occurring during the latent period until completion of host culture lysis. Moreover, comparison with the EMBL-deposited S-PM2d genome (acc. no. LN828717.1) revealed that most of the early mutations were also found in the EMBL-deposited sequence. While this is another testament to the selective advantages of these mutations, considering that the EMBL-deposited genome sequence resulted from 10 years of routine passage of the cyanophage through *Synechococcus* sp. WH7803 [14], it also signifies that the plaque selected for phage stock propagation happened to carry a different set of alleles and/or that the propagation conditions initially selected for these different alleles.

### Later in the *Synechococcus*–S-PM2d coexistence

After a period of host-phage coexistence (ca. day 28–93) characterized by high phage densities despite host population recovery after infection, there appeared to be a shift in the population dynamics around approximately day 134 (~15 bacterial generations; Fig 1). The onset of this shift was earlier in chemostat 1 (ca. day 70; Fig 1A), but otherwise, all three chemostats experienced a fall in their S-PM2d abundances by an order of magnitude to similar densities as those of *Synechococcus*, averaging at 1.78 ±0.58 x 10^7^ mL^-1^ (n = 3). *Synechococcus* abundances in the chemostats also decreased. By day 282, the mean host abundance in the replicate chemostats had dropped to 3.37 ±2.00 x 10^6^ mL^-1^ (n = 3), approximately one-tenth of the control population. The host-phage population dynamics in chemostat 1 showed slight deviation from the other two replicates. Its phage abundance, in particular, was observed to peak briefly around day 282. Thereafter, both the host and phage populations in all three replicate chemostats remained minimal until the end of the experiment.

At this stage (day 500, ~55 bacterial generations), a total of 30 late-occurring, non-synonymous mutations were detected in the S-PM2d populations. These were in addition to the 33 non-synonymous mutations that occurred earlier (day 39, ~4 bacterial generations). Many of the early mutations were retained through to the end, whereas some, in particular the series of SNPs within the virion structural proteins encoded by S-PM2d089 and S-PM2d090, appeared to have reverted to the original alleles but with no clear pattern across the replicate populations observed. Reversion also occurred for one of the two synonymous early mutations (#18). In contrast to the early mutations, the late mutations were found at a larger range of frequencies and at varying degrees of repeatability (Fig 2). Four were indels and the rest were SNPs. Altogether, they occurred within 28 ORFs (Fig 4B), including those encoding very large hypothetical proteins (S-PM2d081, 088 and 218). Only 10 ORFs had not acquired mutations earlier on day-39 (Fig 3B). The majority (seven out of ten) of the late-mutated ORFs encodes hypothetical proteins with unknown functions. Two of them are *nrdA* and *nrdB* (mutations #43 and #44) that encode subunits of the ribonucleotide reductase A, and the remaining ORF encodes a virion structural protein (S-PM2d086; mutation #8).

Common amongst the late mutations is a lack of repeatability, with many of them found to occur in only one or two out of the three replicate chemostats (Fig 2). The SNP within S-PM2d086 encoding a structural protein (mutation #8), as well as the series of SNPs within the short tail fiber-encoding S-PM2d220 (mutations #57–60), were detected only in the phage population of chemostat 2, at frequencies of 83–89% of sequencing depths 285–331. Another example is the SNP detected within the putative gene *nrdA* (mutation #43), which occurred only in chemostat 3. In the sequence scaffold for the original phage strain, 100% of the 1750 bases or reads aligned to the locus 102,473 was adenine. By day 500 into the experiment, 99% of the aligned 580 bases was guanine, resulting in an amino acid substitution of arginine to glycine. Some of the late mutations were also found at low frequencies, indicating weak selection for these mutations. In the phage population of chemostat 2, three SNPs (mutations #44–46) were detected within the putative gene *nrdB*, occurring at the frequencies of 24, 24 and 37% (of sequencing depths 309, 308 and 308, respectively).

## Discussion

Strong similarities were observed across the three replicate chemostats early in the *Synechococcus* sp. WH7803 and cyanophage S-PM2d coexistence, both in terms of the resulting host-phage population dynamics as well as the genomic changes that emerged. The immediate collapse of *Synechococcus* abundances as accompanied by rapid proliferation of S-PM2d were consistent with our expectation that the original host strain was susceptible to infection by the phage [28,29]. The ensuing swift recovery of host population, however, suggests the presence of phage-resistant cells, possibly even prior to initiation of infection pressure but in scarce numbers, ready to take over the available resources and repopulate the culture. This led to a phase that appeared to be stable host-phage coexistence, characterized by high phage densities despite dominance of presumably resistant host cells. These contradictory observations thus point towards the sustenance of susceptible cells in the host population from which the phage could propagate. Consequently, it is likely that the susceptible cells had a trait that gave them an advantage over the resistant cells. This could also explain for the initial dominance of susceptible cells in the *Synechococcus* population prior to infection. Further examinations through isolation of host cells, verification of phage resistance or susceptibility, and growth experiments would be needed to elucidate the mechanism underlying this coexistence. Nonetheless, it is reasonable to speculate that this trait advantage is manifested in the growth rate, given that reduced growth rate has been demonstrated to be as a trade-off for phage resistance in *Synechococcus* sp. WH7803 [30] as well as in a number of other host-virus systems [31,32].

The high frequencies at which the early phage mutations were detected, compounded by their largely consistent repeatability across replicate chemostats, is a convincing indication of their selective advantages. Given that these mutations were subjected to strong selection upon onset of the host-phage coexistence, we propose that they served to optimize host adsorption at a time when many of the host cells were likely phage-resistant. This is supported by our finding that nearly half (eight out of 18) of the ORFs affected by these early mutations–in contrast to roughly one-fifth (37) of all the 211 ORFs in the genome–were predicted to encode virion structures. These eight ORFs included a baseplate wedge, short tail fibre and an adhesin component. The acquisition of mutations in structural proteins was not unexpected, as numerous studies have ascertained that the phage infectivity trait lies in, amongst others, the adsorption to host receptor and injection of phage nucleic acids into the host [7,33,34]. Previous studies on *Pseudomonas fluorescens* SBW25 –phage ɸ2 coevolution reported significantly higher mutation rate in phage genes encoding a tail fibre protein [6] and three other structural proteins, all of which were hypothesized to be directly involved in host adsorption [5]. For this reason, we also speculate that at least some of the affected hypothetical proteins in our study, especially those with mutations at high frequencies, may have structural or virulence functions. Interestingly, no tail fibre gene was identified as a candidate gene associated with the infectivity phenotype in a past coevolution study [8] between *Synechococcus* sp. WH7803 and the cyanophage RIM8 (72% sequence identity to S-PM2d; blastn). Notably, this was based on genome analysis of three phage isolates from one chemostat, in contrast to our population-level analysis.

Resistance against cyanophage in marine *Synechococcus* is primarily facilitated through alterations affecting the host receptor such that phage adsorption is prevented (i.e., extracellular resistance) [8,35,36], which in principle is complementary to the phage counter-defence mechanism discussed above. Mutations linked to the phage resistance phenotype are often localized within highly variable regions termed as genomic islands [32]. The phenotype also appears to be allocated into multiple loci, allowing mutations in entirely different genes to give resistance to the same phage [8]. This thus lends support to the hypothesis that the coevolutionary potentials of bacteria and phages are not equal. Greater mutational constraint acts on phages, as reflected by the limited ways through which phages can restore infectivity [2,7]. Consequently, observation of parallel evolution in a gene is more common in phages than in their hosts [8,37,38]. To date (cross-checked using the web-based tool PADLOC [39]), the CRISPR-Cas and restriction modification systems are not known to exist in *Synechococcus* sp. WH7803. These intracellular systems are in general rare in cyanobacteria of the genera *Synechococcus* and *Prochlorococcus* [40]. While other mechanisms of intracellular resistance have recently been discovered in these genera, they are associated with resistance against cyanophages with broader host ranges (generalists) [36]. Extracellular resistance, on the other hand, is suggested to be largely associated with specialist cyanophages with narrow host ranges, consistent with our knowledge that S-PM2d infects only a number of strains of *Synechococcus* [13].

After the relatively brief period of host-phage coexistence as discussed above, there was in general a fall in S-PM2d abundance accompanied by a slower but steady decline of *Synechococcus* abundance. Extending our discussion earlier on the possible sustenance of susceptible host cells, the present drop in phage abundance may signify a critical point of depletion of these susceptible host cells due to infection, which may also contribute towards the observed host decline. Our present data shows that the net balance of the antagonistic interactions–between infection pressure and host defence–led to minimal but relatively stable abundances throughout the latter half of the experiment. This may be interpreted as another phase of coexistence, distinct from the previous one. We suspect that extensive phenotype diversification of both the host and phage was occurring during this phase, as seen in the earlier coevolution study on *Synechococcus*–RIM8 [8].

The repeatability of the resulting population dynamics and emerged mutations became less apparent as the *Synechococcus–*S-PM2d co-cultivation progressed on. The abundance data for chemostat 1 showed a few notable deviations and a number of late phage mutations detected in the other chemostats were absent in chemostat 1, but more data is needed to draw a link between the two observations. In contrast to the early mutations, the late mutations occurred at a larger range of frequencies, with many detected in only one or two out of the three chemostats. This reflects a capacity for divergence in the evolutionary trajectory with time, which would be consistent with the observation of substantial between-population divergence in resistance and infectivity profiles in the *Pseudomonas fluorescens* SBW25 –ɸ2 system [1]. The trade-off or fitness cost associated with a mutation may play a role in determining the trajectory. Low fitness cost is a selective advantage as it allows a mutation to be acquired and fixed more readily. Thus, earlier in the evolution, mutations with lower fitness costs would be selected over those with higher costs and the same mutations should be acquired repeatedly and independently under replicate conditions. Over time, as more and more of those with lower fitness costs have been taken up, leaving behind a pool of potential mutations with higher costs, stochasticity plays a greater role in determining which of these costly mutations would be acquired. The same argument also applies for a few of the early but less repeatable mutations observed in our study, such as those within auxiliary metabolic genes. These include *nrdC1* which encodes a homolog of glutaredoxin, a small redox enzyme known to be involved in the growth cycle of viruses within their hosts [41,42]. It is probable that such an alteration may affect an essential function and thus have a heavier trade-off. The type of mutation also matters. A single nucleotide insertion within the gene *hli1* for a high-light inducible protein was expected to cause a shift in the downstream reading frame and potentially extensive modifications to the protein and function. The mutation was found in only one chemostat and at relatively low frequency.

Only two synonymous point mutations were detected in our analysis. While these substitutions should not cause amino acid change, it is worth noting that synonymous mutations are known to be capable of altering mRNA stability and translation efficiency, with cascading effects that impact fitness [43]. Rare prevalence of synonymous mutations relative to non-synonymous ones (i.e. high K_a_/K_s_ ratio) is an indication of strong positive selection [44], and was also observed in previous coevolution experiments between *Pseudomonas fluorescens* SBW25 and phage ɸ2 [5,6] and between *Synechococcus* sp. WH7803 and cyanophage RIM8 [8].

## Conclusions

The emergence of an assemblage of strongly selected genomic changes, repeatedly across replicate chemostats, points towards parallel evolution in cyanophage S-PM2d populations early in their antagonistic interactions with *Synechococcus* sp. WH7803. However, it is also evident from our analysis that there was capacity for between-population divergence in their evolutionary trajectory over time. We hypothesize that trade-offs, such as the fitness costs associated with mutations, play a significant role in modulating this trajectory and propose follow-up studies to assess this. Characterization of resistance and infectivity profiles of the host and phage over the course of the experiment is required to validate a coevolutionary relationship and arms-race dynamics presumed here. The study would also benefit from additional phage evolution control, whereby the phage is allowed to evolve but not the host, to further distinguish genomic changes arising through persistent reciprocal interaction with the host (i.e., coevolution) from those attributable to adaptation to the host. Identification of mutations within virion structures associated with adsorption onto host, including tail fibre proteins, challenges the absence of such finding in previous study on marine *Synechococcus–*cyanophage coevolution. This highlights the need within experimental evolution for multiple, complementary approaches to compensate for the limitations of each in disentangling complex processes of the natural environment.

## Supporting information

S1 Appendix Amounts of sequence reads, phage mutations, optical density & phycoerythrin fluorescence, plaque assay. (PDF)

S2 Appendix Supplementary data. (XLSX)
