# Supplementary material for: Molecular evidence of parallel evolution in a cyanophage
Source: PLoS One. 2023 Feb 9;18(2):e0281537. doi: 10.1371/journal.pone.0281537 (PMC9910659; doi:10.1371/journal.pone.0281537)
Supplement: S1 Appendix — (PDF) [file pone.0281537.s001.pdf]

## S1 Appendix

*Amounts of sequence reads*

Table S1-1. Amounts of sequence reads (total raw; aligned 1x, >1x, 0x), as well as the average sequencing depth across all loci in the alignment, from samples of cyanophage S-PM2d populations. R1=forward reads; R2=reverse reads; SD=standard deviation.

| | | Raw Reads | Aligned 1x | Sequencing Depth (mean ± SD) | Aligned 0x | Aligned >1x |
|---|---|---|---|---|---|---|
| Original phage strain | R1 | 3,546,258 | 1,234,064 | 1654 ± 529 | 2,312,116 | 0 |
| | R2 | 3,546,258 | 1,224,223 | | 2,321,956 | 1 |
| Day 39, chemostat 1 | R1 | 2,543,543 | 2,526,905 | 3369 ± 779 | 16,579 | 1 |
| | R2 | 2,543,543 | 2,506,642 | | 36,840 | 2 |
| Day 39, chemostat 2 | R1 | 2,259,626 | 2,239,742 | 2943 ± 666 | 19,815 | 0 |
| | R2 | 2,259,626 | 2,224,509 | | 35,047 | 1 |
| Day 39, chemostat 3 | R1 | 2,652,515 | 2,633,520 | 2950 ± 756 | 18,966 | 0 |
| | R2 | 2,652,515 | 2,615,823 | | 36,660 | 1 |
| Day 500, chemostat 1 | R1 | 3,130,424 | 29,675 | 38 ± 10 | 3,100,666 | 2 |
| | R2 | 3,130,424 | 28,682 | | 3,101,659 | 2 |
| Day 500, chemostat 2 | R1 | 4,919,213 | 250,223 | 311 ± 55 | 4,668,834 | 0 |
| | R2 | 4,919,213 | 246,906 | | 4,672,149 | 0 |
| Day 500, chemostat 3 | R1 | 3,637,675 | 478,641 | 615 ± 175 | 3,158,991 | 0 |
| | R2 | 3,637,675 | 475,105 | | 3,162,522 | 1 |

Note on day-500 samples:

Relative to the total amounts of reads, only very few aligned once to the reference sequence (i.e., consensus sequence generated through alignment of reads from the original phage strain): ca. 0.9, 5 and 13 % for chemostats 1, 2 and 3, respectively. The rest remained unmappable. An attempt to map these reads to the host *Synechococcus* sp. WH7803 sequence (acc. no. CT971583.1) using the same pipeline was not successful either. De novo assembly of these unmappable reads into contigs, however, helped with identification of the source, with the closest match indeed being the host *Synechococcus* sp. WH7803. We suspected that these reads originated from contaminating host cells in the samples. Fewer mapped reads consequently meant lower sequencing depth across the alignment, which in turn may have led to fewer detected mutations, especially towards the ends of the alignment where sequencing depths were generally lowest. However, we deemed the sequencing depths corresponding to the loci of our list of mutations to be sufficient, based on the assessment that a sequencing depth of eight reads that are identical should provide a reliable variant call at an error rate of $10^{-16}$, even if the individual reads have 1% variant-error rate [1,2].

*Phage mutations*

Table S1-2. Mutations in the cyanophage S-PM2d populations, detected early on (day 39) and at the end (day 500) of infection experiment with the host *Synechococcus* sp. WH7803, across three replicate chemostats (C) 1-3. The mutation was either a single nucleotide polymorphism (SNP) within a codon, or an indel (insertion or deletion) that may or may not cause further frameshift within the open reading frame (ORF). The affected nucleotide(s) is underlined. $^{origin}$ refers to the original phage strain, whereas $^{variant}$ refers to the phage population at sampling timepoint (T) of day 39 (early; E) or day 500 (late; L). Shaded rows indicate mutations that were found only once, either on day 39 or day 500 of the experiment. The frequency of the mutation (%), as well as the corresponding sequencing depth (SD), are based on forward read (R1) datasets. AA = amino acid.

| # | ORF | Locus | Mutation | Codon$^{origin}$ | AA$^{origin}$ | %$^{origin}$ (SD) | T | C | Codon$^{variant}$ | AA$^{variant}$ | %$^{variant}$ (SD) |
|---|---|---|---|---|---|---|---|---|---|---|---|
| 1 | S-PM2d064<br>(hypothetical protein, T4-like) | 10,490 | insertion,<br>frameshift | GCA | A (Ala) | 100<br>(4258) | L | 2 | GCA AAC GCC<br>AAG CGT TAC | ANA<br>KRY | 50<br>(568) |
| 2 | S-PM2d073<br>(hypothetical protein, T4-like) | 13,431 | SNP | TGA | * (Ter) | 88<br>(1114) | E | 1, 2, 3 | TAA | * (Ter) | 100, 100, 100<br>(7273, 6329, 6837) |
| | | | | | | | L | | | | 100, 100, 100<br>(79, 540, 1104) |
| 3 | S-PM2d081<br>(hypothetical protein, T4-like) | 27,669 | SNP | AAT | N (Asn) | 95<br>(1271) | E | 1, 2, 3 | GAT | D (Asp) | 100, 100, 100<br>(2973, 2717, 2631) |
| | | | | | | | L | | | | 100, 100, 100<br>(38, 321, 664) |
| 4 | | 27,830 | SNP | AGC | S (Ser) | 100<br>(1243) | L | 3 | AGA | R (Arg) | 98<br>(563) |
| 5 | *gp8*<br>(baseplate wedge) | 29,646 | SNP | TTT | F (Phe) | 72<br>(1229) | E | 1, 2, 3 | TCT | S (Ser) | 100, 100, 100<br>(3249, 2849, 3032) |
| | | | | | | | L | | | | 100, 100, 100<br>(48, 271, 734) |
| 6 | S-PM2d084<br>(virulence associated protein) | 33,115 | SNP | TTT | F (Phe) | 100<br>(1043) | E | 1, 2, 3 | GTT | V (Val) | 100, 100, 100<br>(2909, 2625, 2531) |
| | | | | | | | L | | | | 96, 100, 100<br>(25, 273, 472) |
| 7 | | 35,117 | insertion,<br>no frameshift | TTT | F (Phe) | 100<br>(987) | L | 3 | TTT CTG<br>GAA CTT | FLEL | 83<br>(471) |
| 8 | S-PM2d086<br>(virion structural protein) | 41,264 | SNP | TCG | S (Ser) | 100<br>(975) | L | 2 | TTG | L (Leu) | 88<br>(285) |
| 9 | S-PM2d088<br>(hypothetical protein) | 44,462 | SNP | CTT | L (Leu) | 51<br>(1145) | E | 1, 2, 3 | GTT | V (Val) | 100, 100, 100<br>(3170, 2855, 2879) |
| | | | | | | | L | | | | 100, 100, 100<br>(47, 345, 706) |
| 10 | | 44,831 | SNP | CTA | L (Leu) | 100<br>(1190) | L | 3 | ATA | I (Ile) | 20<br>(610) |
| 11 | | 44,877 | SNP | ACT | T (Thr) | 99<br>(1196) | E | 1, 2, 3 | ATT | I (Ile) | 100, 100, 100<br>(3097, 2799, 2879) |
| | | | | | | | L | | | | 100, 96, 100<br>(36, 278, 677) |
| 12 | | 45,194 | SNP | ATG | M (Met) | 100<br>(1190) | L | 1, 2, 3 | GTG | V (Val) | 81, 98, 66<br>(42, 300, 771) |
| 13 | | 45,196 | SNP | ATG | M (Met) | 100<br>(1190) | L | 3 | GTA | V (Val) | 24<br>(772) |
| 14 | | 48,717 | SNP | GCA | A (Ala) | 100<br>(1552) | L | 3 | GTA | V (Val) | 99<br>(636) |
| 15 | | 49,979 | SNP | TTC | F (Phe) | 100<br>(1300) | L | 2, 3 | CGC | R (Arg) | 93, 99<br>(291, 640) |
| 16 | | 49,980 | SNP | TTC | F (Phe) | 100<br>(1304) | L | 2, 3 | CGC | R (Arg) | 92, 99<br>(290, 638) |
| 17 | | 51,984 | SNP | GGA | G (Gly) | 100<br>(1230) | L | 3 | GAA | E (Glu) | 99<br>(436) |

| | | | | | | | | | | | |
|---|---|---|---|---|---|---|---|---|---|---|---|
| 18 | | 53,840 | SNP | ATA | I (Ile) | 99 (1293) | E | 1, 2, 3 | CTA | I (Ile) | 100, 100, 90 (3236, 2764, 2841) |
| 19 | S-PM2d089 (virion structural protein) | 54,599 | insertion, no frameshift | GGC | G (Gly) | 100 (1213) | L | 1, 3 | GGC GGC CGC CGC | GGRR | 25, 90 (40, 700) |
| 20 | | 54,648 | SNP | CGG | R (Arg) | 100 (1190) | E | 1, 2, 3 | CCG | P (Pro) | 100, 100, 100 (3150, 2865, 2802) |
| | | | | | | | L | | | | 97, 99, 100 (39, 307, 675) |
| 21 | | 54,705 | SNP | AAA | K (Lys) | 100 (1164) | E | 1, 2, 3 | ACA | T (Thr) | 100, 100, 100 (3140, 2818, 2787) |
| | | | | | | | L | 1, 3 | | | 97, 100 (39, 639) |
| 22 | | 54,708 | SNP | AAA | K (Lys) | 100 (1162) | E | 1, 2 | ACA | T (Thr) | 100, 100 (3148, 2806) |
| | | | | | | | L | 1 | | | 97 (38) |
| 23 | | 54,768 | SNP | AAA | K (Lys) | 99 (1168) | E | 1, 2, 3 | AGA | R (Arg) | 100, 100, 100 (3018, 2652, 2649) |
| | | | | | | | L | 1 | | | 97 (35) |
| 24 | | 54,781 | SNP | AAG | K (Lys) | 99 (1157) | E | 2, 3 | AAT | N (Asn) | 72, 100 (2657, 2650) |
| | | | | | | | L | 1, 2 | | | 49, 99 (35, 253) |
| 25 | | 54,785 | SNP | TAT | Y (Tyr) | 99 (1162) | E | 1, 2, 3 | GAT | D (Asp) | 100, 100, 100 2994, 2649, 2622 |
| | | | | | | | L | 1 | | | 97 (33) |
| 26 | | 54,806 | SNP | AAG | K (Lys) | 99 (1135) | E | 1, 2, 3 | GAG | E (Glu) | 100, 100, 100 (2954, 2608, 2591) |
| | | | | | | | L | | | | 97, 98, 100 (32, 250, 517) |
| 27 | | 54,810 | SNP | AAA | K (Lys) | 99 (1135) | E | 1, 2, 3 | ACA | T (Thr) | 100, 100, 100 (2949, 2605, 2577) |
| | | | | | | | L | | | | 97, 100, 100 (31, 255, 509) |
| 28 | | 54,812 | SNP | TCG | S (Ser) | 99 (1142) | E | 1, 2, 3 | GCG | A (Ala) | 100, 100, 100 (2941, 2601, 2564) |
| | | | | | | | L | 1, 3 | | | 97, 78 (30, 501) |
| 29 | | 54,825 | SNP | TTT | F (Phe) | 99 (1135) | E | 1, 2, 3 | TAT | Y (Tyr) | 100, 100, 100 (2931, 2583, 2554) |
| | | | | | | | L | 1, 2 | | | 96, 100 (28, 255) |
| 30 | S-PM2d090 (virion structural protein) | 55,159 | SNP | CCT | P (Pro) | 100 (1184) | L | 1, 3 | CTT | L (Leu) | 62, 99 (26, 468) |
| 31 | | 55,240 | SNP | ACC | T (Thr) | 99 (1232) | E | 1, 2, 3 | AAC | N (Asn) | 100, 100, 100 (2995, 2732, 2644) |
| | | | | | | | L | 1 | | | 100 (24) |
| 32 | | 55,242 | SNP | TTC | F (Phe) | 99 (1237) | E | 1, 2, 3 | GGA | G (Gly) | 100, 100, 100 (3006, 2737, 2644) |
| | | | | | | | L | 1 | | | 100 (24) |
| 33 | | 55,243 | SNP | TTC | F (Phe) | 99 (1233) | E | 1, 2, 3 | GGA | G (Gly) | 100, 100, 100 (3003, 2737, 2648) |
| | | | | | | | L | 1 | | | 100 (24) |
| 34 | | 55,244 | SNP | TTC | F (Phe) | 99 (1234) | E | 1, 2, 3 | GGA | G (Gly) | 100, 100, 100 (3005, 2739, 2651) |
| | | | | | | | L | 1 | | | 96 (24) |
| 35 | | 55,274 | SNP | TTA | L (Leu) | 51 (1280) | E | 1, 2, 3 | TTC | F (Phe) | 100, 100, 100 (3043, 2739, 2667) |
| | | | | | | | L | | | | 100, 100, 100 (23, 297, 384) |

| | | | | | | | | | | | |
|---|---|---|---|---|---|---|---|---|---|---|---|
| 36 | S-PM2d091 | 56,589 | SNP | AGA | R (Arg) | 100 | E | 1, 2, 3 | AGC | S (Ser) | 100, 100, 100 |
| | (virion structural protein) | | | | | (1236) | | | | | (3064, 2706, 2586) |
| | | | | | | | L | | | | 100, 99, 99 |
| | | | | | | | | | | | (25, 270, 308) |
| 37 | S-PM2d117 | 76,808 | SNP | GAA | E (Glu) | 71 | E | 1, 2, 3 | AAA | K (Lys) | 100, 100, 100 |
| | (hypothetical protein) | | | | | (1634) | | | | | (3177, 2736, 2586) |
| | | | | | | | L | | | | 100, 100, 99 |
| | | | | | | | | | | | (43, 344, 578) |
| 38 | | 76,820 | SNP | CCC | P (Pro) | 100 | L | 2 | TCC | S (Ser) | 91 |
| | | | | | | (1647) | | | | | (333) |
| 39 | S-PM2d131 | 87,138 | SNP | GCT | A (Ala) | 99 | L | 3 | GAT | D (Asp) | 99 |
| | (hypothetical protein) | | | | | (1570) | | | | | (780) |
| 40 | S-PM2d132 | 87,809 | SNP | CGC | R (Arg) | 100 | L | 3 | CTC | L (Leu) | 44 |
| | (hypothetical protein) | | | | | (1547) | | | | | (667) |
| 41 | S-PM2d148 | 100,921 | SNP | GTC | V (Val) | 100 | L | 2 | TTC | F (Phe) | 32 |
| | (hypothetical protein) | | | | | (1628) | | | | | (318) |
| 42 | | 100,970 | SNP | GGC | G (Gly) | 100 | L | 3 | GAC | D (Asp) | 42 |
| | | | | | | (1642) | | | | | (666) |
| 43 | *nrdA* | 102,473 | SNP | AGG | R (Arg) | 100 | L | 3 | GGG | G (Gly) | 99 |
| | (ribonucleotide reductase A subunit) | | | | | (1750) | | | | | (580) |
| 44 | *nrdB* | 104,526 | SNP | TCC | S (Ser) | 100 | L | 2 | GAC | D (Asp) | 24 |
| | (ribonucleotide reductase A subunit) | | | | | (1708) | | | | | (309) |
| 45 | | 104,527 | SNP | TCC | S (Ser) | 99 | L | 2 | GAC | D (Asp) | 24 |
| | | | | | | (1708) | | | | | (308) |
| 46 | | 104,532 | SNP | CAA | Q (Gln) | 100 | L | 2 | AAA | K (Lys) | 37 |
| | | | | | | (1713) | | | | | (308) |
| 47 | *nrdC1* | 111,037 | SNP | TGG | W (Trp) | 99 | E | 1, 3 | TGT | C (Cys) | 100, 100 |
| | (glutaredoxin, small redox enzyme) | | | | | (1739) | | | | | (3229, 2938) |
| | | | | | | | L | | | | 50, 99 (42, 697) |
| | S-PM2d163 | | | GTG | V (Val) | | | | TTT | F (Phe) | |
| | (PhDYefM tox-ant domain) | | | | | | | | | | |
| 48 | *nrdC1* | 111,039 | SNP | TGT | C (Cys) | 100 | E | 3 | TTT | F (Phe) | 99 |
| | (glutaredoxin, small redox enzyme) | | | | | (1738) | | | | | (2951) |
| | | | | | | | L | | | | 99 (683) |
| | S-PM2d163 | | | GTG | V (Val) | | | | TTT | F (Phe) | |
| | (PhDYefM tox-ant domain) | | | | | | | | | | |
| 49 | S-PM2d169 | 114,557 | SNP | TTA | L (Leu) | 100 | L | 2 | TAA | * (Ter) | 87 |
| | (hypothetical protein) | | | | | (1804) | | | | | (276) |
| 50 | *hli1* | 114,902 | insertion, | ACC | T (Thr) | 100 | E | 3 | ACC C | T (Thr) | 41 |
| | (high light inducible proteins) | | frameshift | | | (1608) | | | | | (2646) |
| | | | | | | | L | | | | 92 |
| | | | | | | | | | | | (633) |
| 51 | S-PM2d175 | 124,833 | SNP | GAC | D (Asp) | 62 | E | 1, 2, 3 | GAA | E (Glu) | 100, 100, 100 |
| | (virion structural protein) | | | | | (1650) | | | | | (3197, 2733, 2693) |
| | | | | | | | L | | | | 100, 100, 100 |
| | | | | | | | | | | | (53, 324, 639) |
| 52 | S-PM2d186 | 131,751 | SNP | GCT | A (Ala) | 100 | E | 1 | TCT | S (Ser) | 61 |
| | (hypothetical protein) | | | | | (1717) | | | | | (3171) |
| | | | | | | | L | 3 | | | 99 |
| | | | | | | | | | | | (623) |
| 53 | S-PM2d208 | 146,933 | SNP | GAT | D (Asp) | 100 | L | 2 | GGT | G (Gly) | 81 |
| | (hypothetical protein) | | | | | (1890) | | | | | (344) |
| 54 | S-PM2d218 | 159,270 | SNP | TGG | W (Trp) | 100 | L | 3 | CGG | R (Arg) | 99 |
| | (hypothetical protein) | | | | | (1838) | | | | | (524) |
| 55 | | 163,883 | deletion, | TCC GAT GAC | S D D | 86 | E | 1, 2, 3 | TCC | S (Ser) | 89, 91, 89 |
| | | | no frameshift | | | (1736) | | | | | (3269, 2772, 2901) |
| | | | | | | | L | | | | 89, 88, 90 |
| | | | | | | | | | | | (37, 289, 664) |
| 56 | S-PM2d219 | 168,430 | SNP | AAA | K (Lys) | 100 | E | 1, 2, 3 | ACA | T (Thr) | 100, 100, 100 |
| | (short tail fiber) | (168,436) | | | | (1665) | | | | | (3294, 2936, 3006) |
| | | | | | | | L | 1, 2, 3 | | | 100, 100, 30 |
| | | | | | | | | | | | (49, 306, 717) |
| | | | | | | | L | 3 | AGA | | 69 (717) |
| 57 | | 168,447 | SNP | GAA | E (Glu) | 100 | L | 2 | AAA | K (Lys) | 89 |
| | | (168,453) | | | | (1643) | | | | | (309) |
| 58 | | 168,507 | SNP | GTA | V (Val) | 100 | L | 2 | TTA | L (Leu) | 87 |

| | | | | | | | | | | | |
|---|---|---|---|---|---|---|---|---|---|---|---|
| | | (168,513) | | | | (1595) | | | | | (322) |
| 59 | | 168,530 | SNP | AAT | N (Asn) | 100 | L | 2 | AAA | K (Lys) | 88 |
| | | (168,536) | | | | (1627) | | | | | (331) |
| 60 | | 168,537 | SNP | GTT | V (Val) | 100 | L | 2 | ATT | I (Ile) | 83 |
| | | (168,543) | | | | (1633) | | | | | (329) |
| 61 | S-PM2d221 | 170,185 | insertion, | GGT | G (Gly) | 100 | L | 3 | GGT AAT | GN | 83 |
| | (hypothetical protein) | (170,191) | no frameshift | | | (1713) | | | CGA TTA CGT | RLR | (682) |
| 62 | S-PM2d222 | 172,568 | deletion, | ACG | T (Thr) | 100 | E | 2 | ACG | T (Thr) | 92 |
| | (virion structural protein) | (172,574) | frameshift | (ACG GTT A) | | (1612) | | | (ACG TTA) | | (2798) |
| | | | | | | | L | 1, 2 | | | 44, 95 |
| | | | | | | | | | | | (16, 302) |
| 63 | S-PM2d224 | 177,699 | SNP | AAT | N (Asn) | 99 | E | 1, 2, 3 | ACT | T (Thr) | 100, 100, 100 |
| | (short tail fiber) | (177,705) | | | | (1762) | | | | | (3309, 3047, 3100) |
| | | | | | | | L | | | | 98, 100, 100 |
| | | | | | | | | | | | (45, 327, 669) |
| 64 | | 177,707 | SNP | AGT | S (Ser) | 100 | E | 1, 2, 3 | GGT | G (Gly) | 100, 100, 100 |
| | | (177,713) | | | | (1764) | | | | | (3275, 3027, 3100) |
| | | | | | | | L | | | | 98, 100, 37 |
| | | | | | | | | | | | (43, 342, 658) |
| 65 | S-PM2d230 | 181,581 | SNP | TTT | F (Phe) | 100 | E | 1, 2, 3 | GTT | V (Val) | 100, 100, 100 |
| | (hypothetical protein) | (181,587) | | | | (1908) | | | | | (3012, 2697, 2643) |
| | | | | | | | L | | | | 100, 100, 100 |
| | | | | | | | | | | | (33, 307, 523) |

Table S1-3. The frequency, in terms of amount of reads and % of sequencing depth (SD), of each of the four alternative alleles found in the original phage strain for the mutated loci. $SD^{original}$ = sequencing depth of the locus for the original phage strain. A = adenine, C = cytosine, G = guanine, T = thymine.

| | ORF | | Locus | $SD^{original}$ | Amount of reads (% of SD) | | | |
|---|---|---|---|---|---|---|---|---|
| | | | | | A | C | G | T |
| 1 | S-PM2d064 | hypothetical protein, T4-like | 10,490 | 4258 | | | | |
| 2 | S-PM2d073 | hypothetical protein, T4-like | 13,431 | 1114 | 133 (12) | 4 (0) | 977 (88) | 0 (0) |
| 3 | S-PM2d081 | hypothetical protein, T4-like | 27,669 | 1271 | 1212 (95) | 0 (0) | 58 (5) | 1(0) |
| 4 | | | 27,830 | 1243 | 1 (0) | 1241 (100) | 0 (0) | 1 (0) |
| 5 | gp8 | baseplate wedge | 29,646 | 1229 | 0 (0) | 347 (28) | 1 (0) | 881 (72) |
| 6 | S-PM2d084 | virulence associated protein | 33,115 | 1043 | 1 (0) | 0 (0) | 4 (0) | 1038 (100) |
| 7 | | | 35,117 | | | | | |
| 8 | S-PM2d086 | virion structural protein | 41,264 | 975 | 2 (0) | 972 (100) | 1 (0) | 0 (0) |
| 9 | S-PM2d088 | hypothetical protein | 44,462 | 1145 | 2 (0) | 580 (51) | 559 (49) | 4 (0) |
| 10 | | | 44,831 | 1190 | 1 (0) | 1187 (100) | 1 (0) | 1 (0) |
| 11 | | | 44,877 | 1196 | 2 (0) | 1187 (99) | 0 (0) | 7 (1) |
| 12 | | | 45,194 | 1190 | 1189 (100) | 0 (0) | 1 (0) | 0 (0) |
| 13 | | | 45,196 | 1201 | 1 (0) | 1 (0) | 1198 (100) | 1 (0) |
| 14 | | | 48,717 | 1552 | 0 (0) | 1551 (100) | 0 (0) | 1 (0) |
| 15 | | | 49,979 | 1300 | 2 (0) | 1 (0) | 1 (0) | 1296 (100) |
| 16 | | | 49,980 | 1304 | 0 (0) | 2 (0) | 1 (0) | 1301 (100) |
| 17 | | | 51,984 | 1230 | 1 (0) | 0 (0) | 1227 (100) | 2 (0) |
| 18 | | | 53,840 | 1293 | 1285 (99) | 7 (1) | 0 (0) | 1 (0) |
| 19 | S-PM2d089 | virion structural protein | 54,648 | 1190 | 0 (0) | 3 (0) | 1185 (100) | 2 (0) |
| 20 | | | 54,599 | | | | | |
| 21 | | | 54,705 | 1164 | 1161 (100) | 3 (0) | 0 (0) | 0 (0) |
| 22 | | | 54,708 | 1162 | 1159 (100) | 3 (0) | 0 (0) | 0 (0) |
| 23 | | | 54,768 | 1168 | 1161 (99) | 3 (0) | 3 (0) | 1 (0) |
| 24 | | | 54,781 | 1157 | 3 (0) | 0 (0) | 1149 (99) | 5 (0) |
| 25 | | | 54,785 | 1162 | 3 (0) | 0 (0) | 7 (1) | 1152 (99) |
| 26 | | | 54,806 | 1135 | 1127 (99) | 2 (0) | 6 (1) | 0 (0) |
| 27 | | | 54,810 | 1135 | 1124 (99) | 9 (1) | 0 (0) | 2 (0) |
| 28 | | | 54,812 | 1142 | 4 (0) | 0 (0) | 8 (1) | 1130 (99) |
| 29 | | | 54,825 | 1135 | 5 (0) | 0 (0) | 2 (0) | 1128 (99) |
| 30 | S-PM2d090 | virion structural protein | 55,159 | 1184 | 1 (0) | 1181 (100) | 0 (0) | 2 (0) |
| 31 | | | 55,240 | 1232 | 7 (1) | 1225 (99) | 0 (0) | 0 (0) |
| 32 | | | 55,242 | 1237 | 0 (0) | 0 (0) | 7 (1) | 1230 (99) |

| | | | | | | | | |
|---|---|---|---|---|---|---|---|---|
| 33 | | | 55,243 | 1233 | 0 (0) | 1 (0) | 6 (0) | 1226 (99) |
| 34 | | | 55,244 | 1234 | 7 (1) | 1227 (99) | 0 (0) | 0 (0) |
| 35 | | | 55,274 | 1280 | 653 (51) | 625 (49) | 0 (0) | 2 (0) |
| 36 | S-PM2d091 | virion structural protein | 56,589 | 1236 | 1232 (100) | 4 (0) | 0 (0) | 0 (0) |
| 37 | S-PM2d117 | hypothetical protein | 76,808 | 1634 | 464 (28) | 2 (0) | 1167 (71) | 1 (0) |
| 38 | | | 76,820 | 1647 | 4 (0) | 1643 (100) | 0 (0) | 0 (0) |
| 39 | S-PM2d131 | hypothetical protein | 87,138 | 1570 | 3 (0) | 1560 (99) | 3 (0) | 4 (0) |
| 40 | S-PM2d132 | hypothetical protein | 87,809 | 1547 | 3 (0) | 0 (0) | 1541 (100) | 3 (0) |
| 41 | S-PM2d148 | hypothetical protein | 100,921 | 1628 | 0 (0) | 0 (0) | 1626 (100) | 2 (0) |
| 42 | | | 100,970 | 1642 | 2 (0) | 0 (0) | 1638 (100) | 2 (0) |
| 43 | NrdA | ribonucleotide reductase A subunit | 102,473 | 1750 | 1747 (100) | 2 (0) | 1 (0) | 0 (0) |
| 44 | NrdB | ribonucleotide reductase A subunit | 104,526 | 1708 | 1 (0) | 1 (0) | 4 (0) | 1702 (100) |
| 45 | | | 104,527 | 1708 | 9 (1) | 1695 (99) | 1 (0) | 3 (0) |
| 46 | | | 104,532 | 1713 | 5 (0) | 1706 (100) | 0 (0) | 2 (0) |
| 47 | NrdC1 | glutaredoxin, small redox enzyme | 111,037 | 1739 | 1 (0) | 0 (0) | 1729 (99) | 9 (1) |
| | S-PM2d163 | PhDYefM tox-ant domain | | | | | | |
| 48 | NrdC1 | glutaredoxin, small redox enzyme | 111,039 | 1738 | 0 (0) | 0 (0) | 1730 (100) | 7 (0) |
| | S-PM2d163 | PhDYefM tox-ant domain | | | | | | |
| 49 | S-PM2d169 | hypothetical protein | 114,557 | 1804 | 0 (0) | 0 (0) | 1 (0) | 1803 (100) |
| 50 | hli1 | high light inducible proteins | 114,902 | | | | | |
| 51 | S-PM2d175 | virion structural protein | 124,833 | 1650 | 622 (38) | 1027 (62) | 0 (0) | 1 (0) |
| 52 | S-PM2d186 | hypothetical protein | 131,751 | 1717 | 2 (0) | 1 (0) | 1711 (100) | 3 (0) |
| 53 | S-PM2d208 | hypothetical protein | 146,933 | 1890 | 1887 (100) | 1 (0) | 1 (0) | 1 (0) |
| 54 | S-PM2d218 | hypothetical protein | 159,270 | 1838 | 3 (0) | 1 (0) | 2 (0) | 1832 (100) |
| 55 | | | 163,883 | | | | | |
| 56 | S-PM2d219 | short tail fiber | 168,430 (168,436) | 1665 | 1657 (100) | 7 (0) | 1 (0) | 0 (0) |
| 57 | | | 168,453 (168,447) | 1643 | 0 (0) | 1 (0) | 1642 (100) | 0 (0) |
| 58 | | | 168,513 (168,507) | 1595 | 0 (0) | 0 (0) | 1593 (100) | 2 (0) |
| 59 | | | 168,536 (168,530) | 1627 | 0 (0) | 1 (0) | 5 (0) | 1621 (100) |
| 60 | | | 168,543 (168,537) | 1633 | 0 (0) | 1 (0) | 1631 (100) | 1 (0) |
| 61 | S-PM2d221 | hypothetical protein | 170,191 (170,185) | | | | | |
| 62 | S-PM2d222 | virion structural protein | 172,568 (172,574) | | | | | |
| 63 | S-PM2d224 | short tail fiber | 177,699 (177,705) | 1762 | 1743 (99) | 19 (1) | 0 (0) | 0 (0) |
| 64 | | | 177,707 (177,713) | 1764 | 1759 (100) | 3 (0) | 2 (0) | 0 (0) |
| 65 | S-PM2d230 | hypothetical protein | 181,581 (181,587) | 1908 | 1 (0) | 1 (0) | 6 (0) | 1900 (100) |

*Optical density and phycoerythrin fluorescence*

The host population was also monitored through measurements of optical density (OD) at 750 nm [3] and phycoerythrin fluorescence (excitation at 544 nm and emission at 560 nm), using a PerkinElmer® EnSpire™ 2300 Multilabel Reader. Data on the OD of chemostat culture at 750 nm (Fig S1-1) shows similar patterns of *Synechococcus* growth as the FCM data does (Fig 1). The OD of the culture in the control chemostat fluctuated around the mean of 0.04 ±0.009 (n=43), 17 days before phage addition and 317 days onwards. The lowest OD was recorded on day-16 (0.004 ±0.0009, n=3), matching the lowest FCM count recorded (4.83 ±1.31 x $10^5$ $mL^{-1}$, n=3), after which the OD steadily increased and was no longer found to be significantly lower than the OD of the control culture at day-46 (unequal variances *t*-test, p=0.13). The fluctuations displayed by the OD data deviated from the fluctuations in FCM data, but the OD data sufficiently captured key growth transitions of *Synechococcus* in coevolution with S-PM2d, including the population collapse at day-282 (unequal variances *t*-test, p=0.002). Measurement of OD at 750 nm was thus a quick, reliable method for monitoring and roughly estimating *Synechococcus* biomass.

Phycoerythrin is the main light-harvesting pigment in *Synechococcus* sp. WH7803 [4]. Data on phycoerythrin fluorescence (excitation at 544 nm, emission at 560 nm) may shed some light on the ‘photosynthetic’ activity of the *Synechococcus* – S-PM2d culture. Within four days of phage infection, phycoerythrin fluorescence increased sharply to reach a peak

almost six times the intensity as the control (Fig S1-2). This observation is consistent with an earlier finding based on quantitative real-time PCR that S-PM2 increased the transcription levels of the phycoerythrin-encoding operons *mpeBA* and *cpeBA* 46- and 22-fold, respectively, within 9 hours of infection [5]. The same study concluded that this surge in light-harvesting capacity and, presumably, photosynthetic activity may be a strategy to meet the energy demand of phage proliferation. The fluorescence dropped drastically thereafter to the lowest recorded mean on day-16, which corresponded with the lowest *Synechococcus* FCM count and $OD_{750\ nm}$. This is congruent with a period of host lysis. By day-28 and onwards until at least day-317 of the coevolution, phycoerythrin fluorescence was not found to differ from the control due to S-PM2d infection.

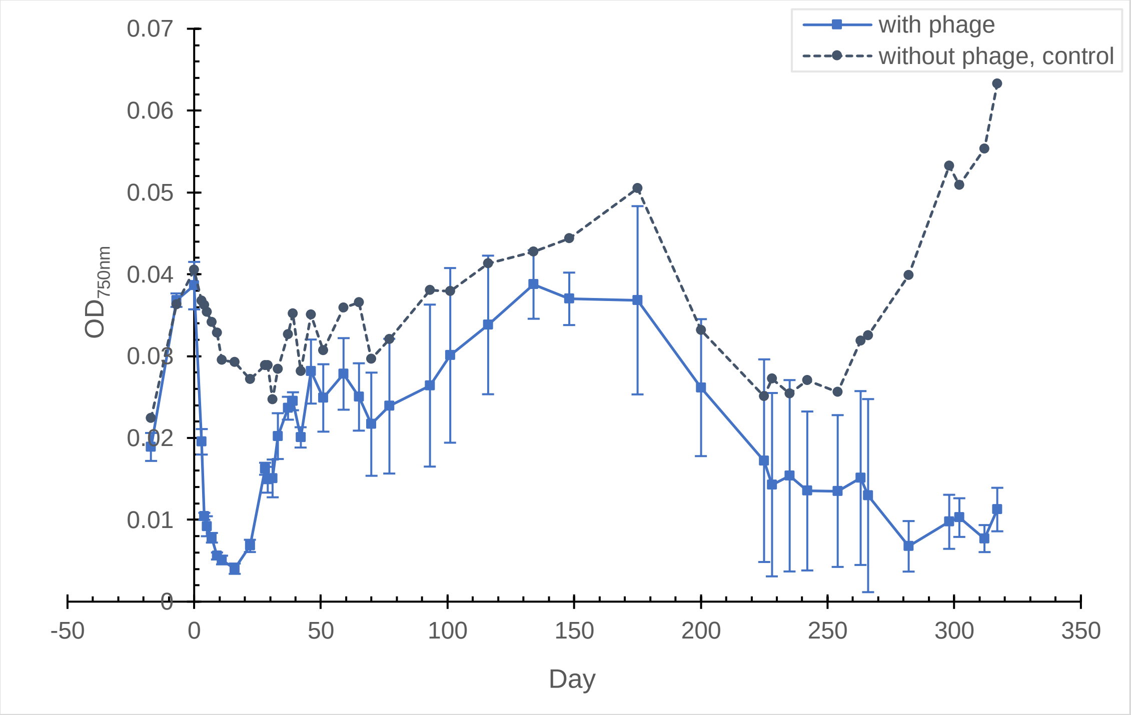


Fig S1-1. The optical density at 750 nm ($OD_{750nm}$) of the host *Synechococcus* sp. WH7803 in coevolution with the phage S-PM2d (added at day-0). Except for the control, plotted values were averaged from three replicates (n=3). Error bar = SE of the mean.

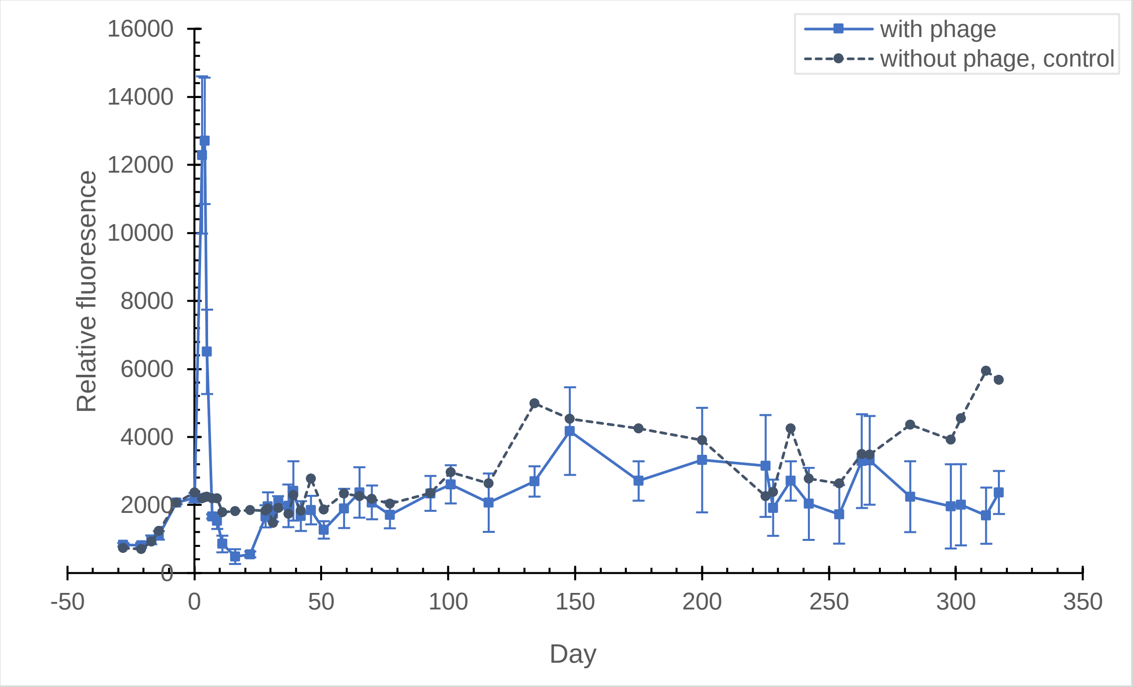


Fig S1-2. Phycoerythrin fluorescence (excitation at 544 nm, emission at 560 nm) of the host *Synechococcus* sp. WH7803 in coevolution with the phage S-PM2d (added at day-0). Except for the control, plotted values were averaged from three replicates (n=3). Error bar = SE of the mean.

### *Plaque assay*

Flow cytometry (FCM) has its merits in enabling a higher throughput and precise monitoring of abundances in this experiment. This is especially useful for phage enumeration due to fewer alternatives. FCM is, however, limited to the counting of virions and does not directly provide any data on the infectivity of these virions. Plaque forming units (PFU), as a measure of phage concentration that could still infect the original host strain, was determined 65 days (~7 generations) following phage addition. The plaque assay was done by centrifuging a 50 mL culture of *Synechococcus* sp. WH7803 at 6000 rpm for 20 min, removing the supernatant and re-suspending the cells in approximately 5 mL ASW, to make a dense culture. Dilution series of S-PM2d sampled from the initial culture and from the chemostats were made, and 10 µL from each dilution was added to 500 µL of the dense host culture. The mixture was then incubated for 15 min at 23 °C and under continuous light (20 - 23 µE $m^{-2}$ $s^{-1}$) to allow adsorption of the phage to the host. The mixture was then mixed with 3 mL 0.4 % w/v overlay (ASW) agar and poured onto an ASW agar plate. Solidified plates were incubated (23 °C, continuous light) for 2 - 4 weeks to allow plaques to form.

A plaque assay on S-PM2d sampled from the chemostats at day-65 revealed an infectious abundance of 3.85 ±1.79 x $10^6$ $mL^{-1}$ (n=3), which corresponds to 1.5% of the virion count (2.62 ±1.09 x $10^8$ $mL^{-1}$, n=3).

*References*


1. Schatz MC, Delcher AL, Salzberg SL. Assembly of large genomes using second-generation sequencing. Genome Res. 2010 Sep;20(9):1165–73. doi: 10.1101/gr.101360.109

2. Sims D, Sudbery I, Ilott NE, Heger A, Ponting CP. Sequencing depth and coverage: Key considerations in genomic analyses. Nat Rev Genet. 2014 Feb;15(2):121–32. doi: 10.1038/nrg3642

3. Wilson WH, Carr NG, Mann NH. The effect of phosphate status on the kinetics of cyanophage infection in the oceanic cyanobacterium *Synechococcus* sp. WH7803. J Phycol. 1996 Aug;32(4):506–16. doi: 10.1111/j.0022-3646.1996.00506.x

4. Ong LJ, Glazer AN. Phycoerythrins of marine unicellular cyanobacteria. I. Bilin types and locations and energy transfer pathways in *Synechococcus* spp. phycoerythrins. J Biol Chem. 1991;266(15):9515–27. doi: 10.1016/s0021-9258(18)92851-6

5. Shan J, Jia Y, Clokie MRJ, Mann NH. Infection by the ‘photosynthetic’ phage S-PM2 induces increased synthesis of phycoerythrin in *Synechococcus* sp. WH7803. FEMS Microbiol Lett. 2008 Jun 28;283(2):154–61. doi: 10.1111/j.1574-6968.2008.01148.x
